# Case Report: Infra-Low-Frequency Neurofeedback for PTSD: A Therapist's Perspective

**DOI:** 10.3389/fnhum.2022.893830

**Published:** 2022-05-24

**Authors:** Regula Spreyermann

**Affiliations:** Praxis Dr. med. Regula Spreyermann, Basel, Switzerland

**Keywords:** post-traumatic stress disorder (PTSD), neurofeedback for PTSD, neurofeedback combined with psychotherapy, hyperarousal, trauma, C-PTSD

## Abstract

The practical use of a combination of trauma psychotherapy and neurofeedback [infra-low-frequency (ILF) neurofeedback and alpha-theta training] is described for the treatment of patients diagnosed with complex post-traumatic stress disorder (C-PTSD). The indication for this combined treatment is the persistence of symptoms of a hyper-aroused state, anxiety, and sleep disorders even with adequate trauma-focused psychotherapy and supportive medication, according to the Guidelines of the German Society of Psycho-Traumatology (DeGPT). Another indication for a supplementary treatment with neurofeedback is the persistence of dissociative symptoms. Last but not least, the neurofeedback treatment after a trauma-focused psychotherapy session helps to calm the trauma-related reactions and to process the memories. The process of the combined therapy is described and illustrated using two representative case reports. Overall, a rather satisfying result of this outpatient treatment program can be seen in the qualitative appraisal of 7 years of practical application.

## Setting For The Treatment Of Patients With Complex Post-Traumatic Stress Disorder Using Trauma-Focused Psychotherapy And Neurofeedback

For indications such as complex post-traumatic stress disorder (C-PTSD), a close interdisciplinary collaboration is called for. In this case, a close collaboration between the psychiatric doctor's office with a focus on psycho-traumatology (Peter Streb, MD, Psychiatry and Psychotherapy, Basel, Switzerland) and the office for psychosomatic medicine offers neurofeedback (Regula Spreyermann, MD Internal Medicine, Basel, Switzerland).

In trauma-focused psychotherapy, Peter Streb works according to the Guidelines of the DeGPT, the German Society of Psychotraumatology (https://www.awmf.org) with stabilization, trauma confrontation, and processing including specific interventions such as Eye Movement Desensitization and Reprocessing (EMDR) or Imagery Rescripting and Reprocessing Therapy (IRRT).

The additional psychosomatic treatment includes psychoeducation, neurofeedback, mindfulness training, imaginative therapy, and general support throughout the process and with optimization of the conditions of living (Lake, 2015).

Once a week, the effects of the combined therapy, the condition of the patients, acute problems, medication changes, and the overall process are discussed interdisciplinary between the doctor's offices. Based on this regular exchange, decisions are made with respect to changes in training protocol or shifting the focus to either more psychotherapy or more neurofeedback or only on one of them. This happens either because the objective has been reached or because it became clear that the additional effect is insufficient.

## Indications to Add Neurofeedback to Trauma-Focused Psychotherapy in the Treatment of Patients Suffering From C-PTSD

Patients are sent to an additional treatment with neurofeedback when, despite intensive trauma-specific psychotherapy and different medications, insufficient relief from the typical PTSD symptoms has been achieved (van der Kolk et al., 2016). In these cases, infra-low-frequency (ILF) neurofeedback (Othmer, 2017), followed by alpha-theta training (Othmer and Othmer, 2017), is used as an additional treatment of choice (Lanius et al., 2015).

There are also cases where a trauma-induced chronic dissociation hinders effective psycho-therapeutic trauma therapy, as emotions cannot be perceived by the patient (Lanius and Frewen, 2015). In these cases, neurofeedback can help to lead to better self-awareness, greater mental stability, and improved self-regulation, which then in turn makes it possible to work on the trauma (Gerge, 2020a).

## PTSD Symptoms and What Makes it C-PTSD

The typical symptoms of PTSD are symptoms of chronic stress induced by trauma. These symptoms include hyperarousal, sleep disorders, panic attacks, nightmares, flashbacks, muscle tension, fatigue, lack of concentration, emotional instability, and depressive symptoms. One speaks of C-PTSD if the persistent PTSD symptomatology has led additionally to personality changes and emotional dysregulation according to the criteria of the International Classification of Diseases 11th Revision (World Health Organization, 2022), which are typically induced by persistent traumatization during childhood (emotional or sexual abuse or violence or neglect) or during adulthood following torture, abuse, violence, or loss. The persons affected show symptoms of constant hyperarousal of the stress system with inner unrest, anxiety, panic, sleep disorders, nightmares, exhaustion, depression, and obsessive-compulsive behavior. There are also physical symptoms of chronic tension that are chronic pain in the musculoskeletal system, bruxism, dental defects, and headache (Cloitre et al., 2013). Due to disorders of the immune system caused by an impaired release of cortisol, there is a high prevalence of infections, irritable bowel, and other conditions (Boscarino, 2009). As a consequence of the physical and mental exhaustion, ADHD-like symptoms (Kimbrel et al., 2017) such as distractibility, concentration disorders, or procrastination can occur, which additionally can have an effect on working capacity. The presence of trauma, flashbacks, and avoidance behavior confirms the PTSD diagnosis.

The personality changes that lead to the diagnosis of a C-PTSD are negative thoughts about themselves and others, mistrust, and avoidance of social contacts.

The criteria for the diagnosis according to ICD/DSM are adapted over time. A current discussion is the concept of developmental traumatization. If the trauma is severe and diagnosed at a late stage, in most cases, specialized psychotherapy and usual medicinal treatment are required. Neurofeedback offers in this study a promising additional benefit (van der Kolk et al., 2020; Micoulaud-Franchi et al., 2021).

## Practical Steps in the Treatment of Patients With C-PTSD

The first step in the whole process takes place in the psychotherapy practice. There is the need for a proper first interview to gain a perspective on the history and background of the patient and to determine which problems have priority. It may be necessary to react with pharmacotherapy in the first instance (sleep medicine, antidepressants, or anxiolytics), or to initiate help with acute psychosocial problems by contacting other doctors, family, employers, or insurance. In a further step, it is important to move deeper into the special trauma therapeutic methods such as Imagery Reprocessing and Rescripting therapy IRRT (Grunert et al., 2007) or Eye Movement Desensitization and Reprocessing EMDR (Shapiro, 1995). However, in many cases, it is necessary to pursue mental (Lanius et al., 2017) and somatic stability as a priority before working on the real trauma, and this can be achieved using the neurofeedback as an additional next step (Panisch and Hang Hai, 2020). So the patients are informed about this possibility and assigned to the psychosomatic therapist to initiate the training (Gerge, 2020b).

The patients are informed about neurofeedback as a method, about gained experiences, and the expected improvement as well the possibility, in which within the first 10–15 sessions ups and downs may occur. Based on this information, the shared decision is made whether neurofeedback therapy is started or not. The patient gets informed, that an evaluation will take place after 10–20 sessions to consider, whether the treatment is sufficiently supportive or not.

In a further step, the medical, the personal, and the family histories are elaborated on, as well as the current symptoms' presentation before we start. The assessment of the individually important symptoms is conducted using a computerized symptom questionnaire by EEG Expert called “Symptomtracking” (https://eegexpert.net). It helps to rate the severity of the relevant symptoms on a scale from 0 to 10. In the course of the neurofeedback treatment, the symptom tracking is repeated every 2–3 months to be able to track the progress, but also to be able to see in which domain more focus has to be placed. The neurofeedback protocol priority is adjusted accordingly (Reiter et al., 2016).

The principal electrode placement used for trauma resolution is T4-P4, which can be complemented or if necessary, changed to T3–T4 in case of instabilities, according to the Protocol Guide 2017 by Sue Othmer. During the initial period of finding, the optimal response frequency (ORF) to address the existing symptoms such as hyperarousal, sleep disorders, flashbacks, nightmares, anxiety, or muscle tensions, especially in patients with high instability, ups and downs, and undesired side effects can occur during the session, or within hours or days after the session. These effects mostly last only for a few hours up to 1 or 2 days, and they help us to find the ORF (Wiedemann, 2020).

After the ORF is found, it is important to find an optimal rhythm for the sessions. One session per week is typical for an outpatient setting; however, a biweekly rhythm can be better tolerated. Regularity is of great importance, as the training induces a process that ideally should not be interrupted, at least not in the first 2 months.

Within this process, indications for additional sensor placements can arise. The symptom tracking helps to monitor the long-term course and also helps the patient to see progress and stay motivated. Weekly communication with the psychotherapist is of great importance to optimize the process for the patient. When sufficient stabilization of the patient is achieved, trauma confrontation may become easier. In a further step, synchrony protocols (both in the ILF and in the alpha band) may be incorporated into neurofeedback therapy as an additional self-regulation strategy. Synchrony training can also serve as a means to assess the readiness to undertake alpha-theta training to support the psychological reprocessing of the trauma (Imperatori et al., 2017).

## Case Presentations

In the following, two representative cases are elaborated to illustrate the clinical process.

### Case 1

Case 1 is a 40-year-old female suffering from C-PTSD caused by the violent death of her mother when she was around 20 years old. Over the years, there was psychotherapy with various therapists, followed by around 10 years of psycho-trauma therapy. Due to the residual hyperarousal, massive sleep dysregulation, and nightmares, neurofeedback therapy was advised. Sustained daytime flashbacks and dissociation were reported by the patient. In fact, the patient acted entirely emotionless and absent. The answers were purely rational. In contrast to the posture of “not-being-noticeable,” the patient reports massive emotions such as fury, grief, helplessness, anxiety, and panic being dissociated felt for her like dizziness. In addition, she was plagued by massive headaches, stress-induced skin reactions, and tinnitus. The prehistory is heavily loaded, growing up in a family full of conflicts, violence both emotional and physical, a depressive and suicidal father, and a mother full of sorrow about her drug-addicted brother.

There had been three suicides in the immediate family and among close friends—she herself lost a close friend when she was 5 years old, and at the age of 10, someone committed suicide directly in her presence. Since childhood, she suffered from poor sleep, nightmares, and restlessness in sleep. She had daydreams about being adopted and her true parents coming to rescue her. She had been ridiculed at school about her skewed teeth as well as by her father. She withdrew and felt like belonging nowhere. As a teenager, there were suicidal thoughts, binge eating, and many illnesses. In her early 20s, the mother died in violent death, and the father is also now deceased. Anniversaries of the deaths always increase the trauma-induced symptoms, despite the passage of time. Looking at her childhood, there are many consecutive events that led collectively to the severe traumatization.

### Neurofeedback Training and Symptom Tracking

Due to a high emotional instability and very noticeable mistrust and skepticism, T4-P4 was trained in the beginning. This calmed down the patient. However, it also led to heavy side reactions such as mood swings. For this reason, T3-T4 was added after session 7. After 25 sessions, T4-Fp2 was added. This protocol targets the affective domain more directly. Again, there were panic states, which were treated with T3-T4 for a few sessions. Now she is back to a treatment solely with T4-P4 and alpha-theta.

**Figure 1:**
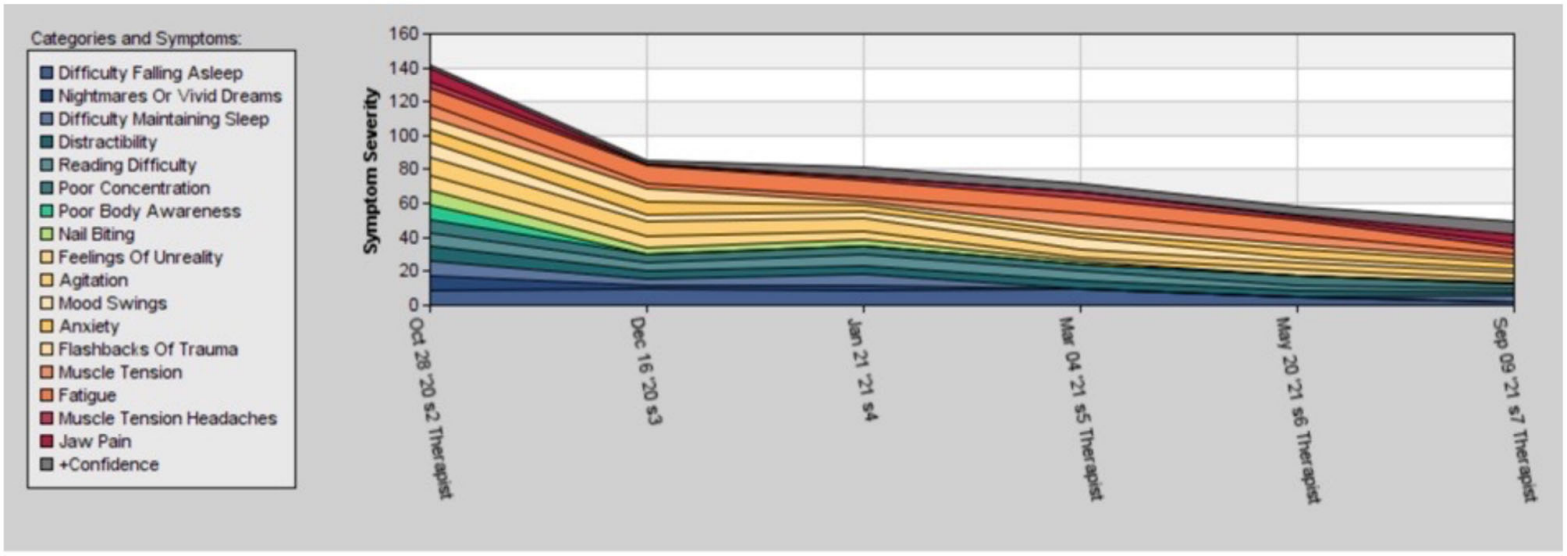
Time course of the different post-traumatic stress disorder (PTSD) symptoms of case 1 during 1 year of therapy, rated by the patient between 0 and 10.

In Figure 1, the symptom tracking over the course of time for the main symptoms is illustrated. The figure shows the steady decline of symptom severity to below 30% of the starting value, over the course of 47 sessions. These symptoms had existed for more than 20 years and could not be eliminated using medication and psychotherapy.

In her own words, the patient describes herself as being back to herself, back to the here and now, experiencing more placidity and equanimity, and much better sleep.

The patient has now found her way back into life. She has finished her dissertation, which she had started with panic attacks and under high stress, without drama. She has found appropriate employment. In the future, there is some work yet to be done in terms of personal relationships. Due to the history of C-PTSD and the ongoing personality changes, there is still a lot of avoidance in her life, especially in terms of intimate relationships.

### Case 2

Case 2 is the story of a 35-year-old female with C-PTSD. The trauma dates back to early childhood. She was adopted as an infant from the Far East by a German family with two children. There was never the feeling of belonging to the family, and this has persisted until the present. Looking Asian while being German, she has not found her real identity. This is one of the reasons that she chose to start neurofeedback. The trauma was triggered by continuous sexual abuse during primary school age, but also from repeated relationships with heavy physical violence by the partner, including brutal rape. As in the first case, the trauma in this study cannot be traced back to a single event, but rather to multiple insults. The patient suffers from permanent and clear recognizable dissociation: her face looks mask-like and her voice is thin and unemotional. There is a steady twitching of her eyelids due to flashbacks from violent scenes coming every few seconds.

She has massive concentration and memory impairment, making it impossible for her to work. Her sleep quality is extremely poor; she awakes every few minutes; falling asleep initially takes several hours. During the day, she is steadily tired and exhausted. The only constant in her life has been her daughter for whom she cares daily, cooks, goes horseback riding, and much more. She has been doing neurofeedback treatment for around 4.5 years now. It stands out since the beginning of the NF training that in combination with psychotherapy, there is a steady and very positive development, one that is not always visible in symptom tracking. The first noticeable improvement was enhanced sleep, which helped her in her daily life. She could deal better with her household and learned how to play golf to enhance her concentration and be more outdoors. After 2 years, she relates that she is less dissociated, “which is not always pleasant.”

In a second step, she was able to reduce the extensive psychiatric medication; first, the neuroleptics, then, the antidepressants, and lastly, the sleeping pills. The only thing left is sleep-inducing antidepressants.

In further progress, there was the separation from her pill-addicted husband, where the relationship had been very destructive. Another relationship ended with a physical attack. After being on her own for several years, she is now in a new relationship. Psychotherapy helps her to learn the fundamentals of a trusting relationship. She has even successfully managed to build up a company of her own.

### ILF Training and Symptom Tracking

**Figure 2:**
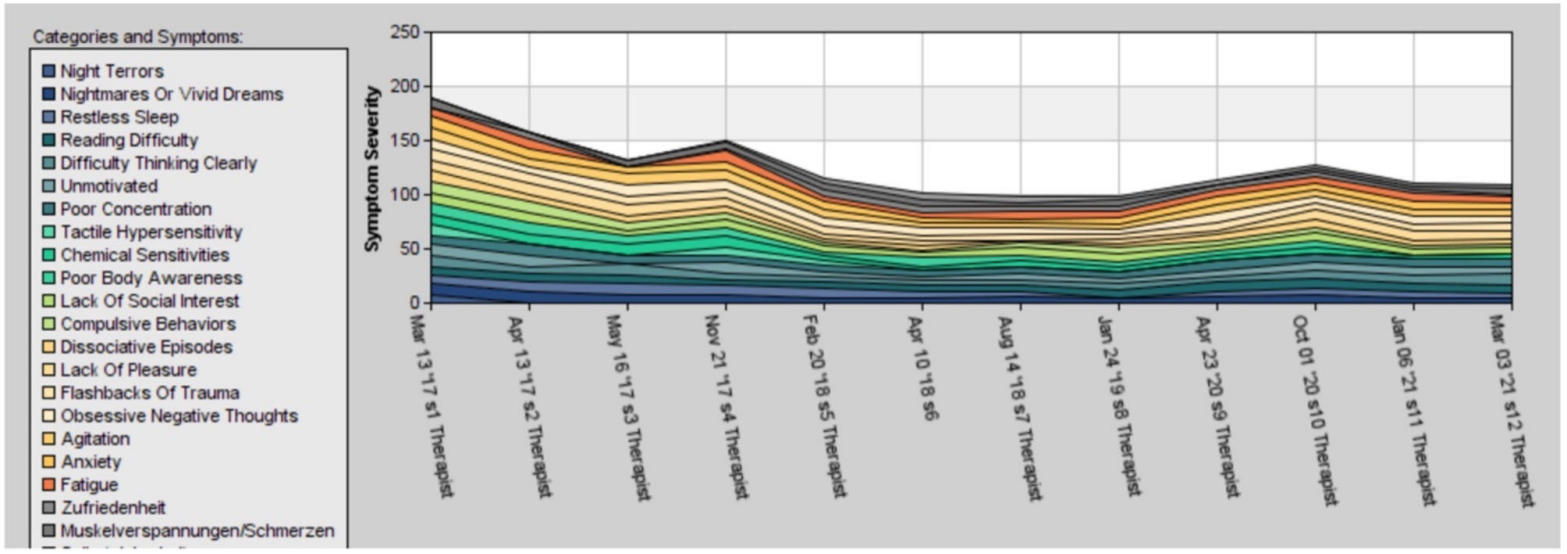
Development of the PTSD symptom during the 5 years of therapy in case 2. The ups and downs are the results of reduction of medicaments, life events such as divorce, and re-traumatization.

The symptom tracking in Figure 2 shows that in the beginning, there is a quick improvement in symptom severity followed by fluctuations. In October 2020, there was a relapse caused by the terminal illness of her father.

The treatment of heavily traumatized patients is not straightforward, as the relationship is often characterized in the beginning by high levels of mistrust, avoidance, and need for control in the frame of the attachment trauma. When sleep improved, she reduced her medication, which led to instabilities and the need to find a new ORF.

For a while, she wanted to be independent and, therefore, stretched the frequency of the meetings to a maximum. She only came when sleep got worse again after around 3-4 weeks. Since there was no further improvement due to the irregular training, more intensive training was resumed. After 2 years, consecutive new placements were added to T4P4, and now training on all four basic sites is possible (T4-P4, T3-T4, T4-Fp2, and T3-Fp1). Fp1-Fp2 training helps to reduce compulsive behavior and racing thoughts.

In her own words, the patient asserts that before the neurofeedback treatment, she had to take the strongest medications and yet was unable to sleep well at all. Now, after 2–3 years of neurofeedback therapy, she sleeps more profoundly for more hours and experiences a better sleep quality. She says that she is no longer afraid of going to bed. In addition, she describes that she feels her body again—where earlier she had a feeling of numbness and her body had not been present in her thoughts.

### An Overall Appraisal of Clinical Results

A quantitative appraisal of clinical effectiveness in the manner of a formal retrospective study is not appropriate, given the heterogeneity of the clinical population, as well as variety of measures taken with each patient. However, looking back qualitatively over the past 7 years, the combination of trauma-based psychotherapy and ILF neurofeedback has led to surprising and motivating results.

For approximately 25% of the patients, there was an initial improvement of the symptoms, but in the course of the therapy, there was a stagnation of progress. In these cases, the addition or adoption of other methods such as mindfulness training, HRV-training, sports, or respiratory therapy should be considered.

In only 2 of 80 patients has there been no positive reaction to the neurofeedback.

For around 15% of the cases, premature abandonment of the therapy was recorded, which can be traced to a variety of reasons, which is common in the domain of psychotherapeutic treatments.

However, for the rest of the patients with C-PTSD, good or very good progress was observed. An improvement of 60% up to over 90% could be seen in symptom tracking, which is very surprising given the severity of the initiating traumas and the long-established symptom history of the patients.

Infra-low-frequency neurofeedback is emerging as a promising method to help patients with PTSD. Both case reports show that people who suffered a great deal and who were not able to live a normal life were able to go back into daily routines with the help of neurofeedback therapy. Even though not all patients benefit, the strong likelihood of a positive outcome is promising. One also must keep in mind that in many cases, patients start neurofeedback therapy after having already suffered a long time and having tried several therapies without success.

## Data Availability Statement

The original contributions presented in the study are included in the article/supplementary material, further inquiries can be directed to the corresponding author/s.

## Ethics Statement

Written informed consent was obtained from the individual(s) for the publication of any potentially identifiable images or data included in this article.

## Author Contributions

The author confirms being the sole contributor of this work and has approved it for publication.

## Funding

Open access fees are covered by the Brian Othmer Foundation, 6400 Canoga Ave., Suite 210, Woodlands Hill, CA 91367.

## Conflict of Interest

The author declares that the research was conducted in the absence of any commercial or financial relationships that could be construed as a potential conflict of interest.

## Publisher's Note

All claims expressed in this article are solely those of the authors and do not necessarily represent those of their affiliated organizations, or those of the publisher, the editors and the reviewers. Any product that may be evaluated in this article, or claim that may be made by its manufacturer, is not guaranteed or endorsed by the publisher.
